# Clinical Features, Diagnosis, and Treatment of COVID-19 in Hospitalized Patients: A Systematic Review of Case Reports and Case Series

**DOI:** 10.3389/fmed.2020.00231

**Published:** 2020-05-15

**Authors:** Azin Tahvildari, Mahta Arbabi, Yeganeh Farsi, Parnian Jamshidi, Saba Hasanzadeh, Tess Moore Calcagno, Mohammad Javad Nasiri, Mehdi Mirsaeidi

**Affiliations:** ^1^Student Research Committee, School of Medicine, Shahid Beheshti University of Medical Sciences, Tehran, Iran; ^2^Department of Microbiology, School of Medicine, Shahid Beheshti University of Medical Sciences, Tehran, Iran; ^3^Division of Pulmonary, Critical Care, Sleep and Allergy, Department of Medicine, Miller School of Medicine, University of Miami, Miami, FL, United States

**Keywords:** COVID-19, clinical characteristics, diagnosis, treatment, systematic review

## Abstract

**Introduction:** The 2019 novel coronavirus (COVID-19) has been declared a public health emergency worldwide. The objective of this systematic review was to characterize the clinical, diagnostic, and treatment characteristics of hospitalized patients presenting with COVID-19.

**Methods:** We conducted a structured search using PubMed/Medline, Embase, and Web of Science to collect both case reports and case series on COVID-19 published up to April 24, 2020. There were no restrictions regarding publication language.

**Results:** Eighty articles were included analyzing a total of 417 patients with a mean age of 48 years. The most common presenting symptom in patients who tested positive for COVID-19 was fever, reported in up to 62% of patients from 82% of the analyzed studies. Other symptoms including rhinorrhea, dizziness, and chills were less frequently reported. Additionally, in studies that reported C-reactive protein (CRP) measurements, a large majority of patients displayed an elevated CRP (60%). Progression to acute respiratory distress syndrome (ARDS) was the most common complication of patients testing positive for COVID-19 (21%). CT images displayed ground-glass opacification (GGO) patterns (80%) as well as bilateral lung involvement (69%). The most commonly used antiviral treatment modalities included, lopinavir (HIV protease inhibitor), arbidiol hydrochloride (influenza fusion inhibitor), and oseltamivir (neuraminidase inhibitor).

**Conclusions:** Development of ARDS may play a role in estimating disease progression and mortality risk. Early detection of elevations in serum CRP, combined with a clinical COVID-19 symptom presentation may be used as a surrogate marker for the presence and severity of the disease. There is a paucity of data surrounding the efficacy of treatments. There is currently not a well-established gold standard therapy for the treatment of diagnosed COVID-19. Further prospective investigations are necessary.

## Introduction

Late in December 2019 and early in January 2020, reports of a very progressive pneumonia-like respiratory syndrome, starting in Wuhan, China, induced global health concerns (1). Soon after the onset of disease, it was found that the pathogen was a new member of the coronaviridae family, named SARS-COV-2 which is now called 2019-n-CoV (2). The respiratory syndrome caused by 2019-n-CoV is called COVID-19. COVID-19 is characterized by low-grade fever, cough, dyspnea, lymphopenia, and ground-glass opacities on chest CT scan (3, 4). COVID-19 is a highly contagious disease, probably an aerosol born one, with human to human transmission capacity which has implicated many countries all around the world (5). In this review article, we systematically surveyed case reports and case series from many countries in the world to give a picture of the epidemiology, clinical presentations, laboratory changes, imaging findings, diagnostic criteria, treatments, outcomes, prognostic factors, and risk factors of COVID-19 in hospitalized patients.

## Methods

This review conforms to the “Preferred Reporting Items for Systematic Reviews and Meta-Analyses” (PRISMA) statement (6).

### Search Strategy

We carried out systematic searches of the literature in the following bibliographical databases: PubMed/Medline, Embase, and Web of Science. Search criteria included case reports and case series articles published up to April 24, 2020, and there were no restrictions regarding publication language. We used Google Translate for eligible articles published in languages other than English. The search terms for our review were: COVID-19, severe acute respiratory syndrome coronavirus 2, novel coronavirus, SARS-CoV-2, nCoV disease, SARS2, COVID-19, 2019-nCoV, coronavirus disease-19, coronavirus disease 2019, and 2019 novel coronavirus.

### Study Selection

Studies included in the review met the following criteria: prospective or retrospective descriptive case reports and case series of COVID-19 in the hospital setting which included diagnostic methods, clinical manifestations, laboratory features, treatment, and outcomes. Articles describing experimental approaches as well as reviews and publications without peer-review processes were excluded.

All potentially relevant articles were screened in two stages for eligibility. In the first stage, the titles and abstracts of potentially relevant articles were screened independently by two reviewers (YF, PJ). In the second stage of assessment, the full text of those abstracts which met the inclusion criteria was retrieved and independently reviewed by the same authors. Disagreements and technical uncertainties were discussed and resolved between review authors (AT, SH, MA, MJN).

### Data Extraction

The extracted data included bibliographic data, patient demographics (e.g., age and gender), radiological and laboratory findings, treatment protocols, and medical consequences. Two authors (AT, SH) independently extracted the data from the selected studies. The data was jointly reconciled, and disagreements were discussed and resolved between review authors (YF, PJ, MA, MJN).

## Quality Assessment

The critical appraisal checklist for case reports provided by the Joanna Briggs Institute (JBI) was used to perform a quality assessment of the studies (7).

## Results

**Figure 1:**
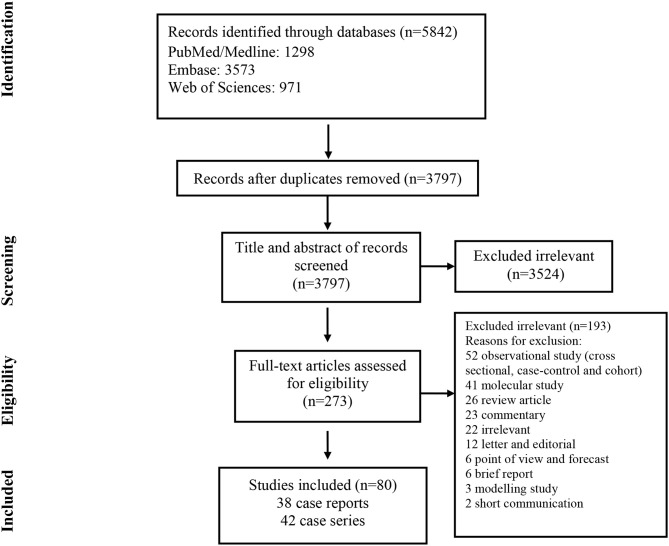
Flow chart of study selection for inclusion in the systematic review.

As illustrated in Figure 1, our systematic search resulted in an initial number of 6,004 of potentially relevant articles, of which 1,033 were excluded by title and abstract evaluation. Applying the inclusion/exclusion criteria to the full-text documents, 80 articles were eligible for inclusion in the systematic review. 42 case reports and 38 case series from 19 countries were identified with a total of 417 unique cases of COVID-19 with a mean age of 48 years (Table 1). The included case reports were published because of the following reasons: they reported (1) new CT findings; (2) new clinical manifestations; (3) new laboratory findings, (4) new treatment outcomes; (5) atypical manifestations and some were the first one in a specific country. Based on the JBI tool, the included studies had a low risk of bias. RT-PCR COVID-19 was present in 79 (95%) articles as inclusion criteria. In addition to RT-PCR, a CT scan served as a diagnostic tool in 16 (19%) of papers. Reported comorbidities included hypertension, diabetes, cardiovascular disease, and pulmonary disease. Hypertension was investigated the most, studied in 22/83 (26.5%) of papers. Of the 16 COVID-19 positive patients found in the studies investigating hypertension, 44 patients were hypertensive (19%) (Table 2). Lymphopenia was reported in 24 studies which identified 83/185 (45%) of COVID-19 positive patients. Additionally, in studies that reported C-reactive protein (CRP) measurements, a large majority of patients displayed an elevated CRP (60%). CT images commonly displayed ground-glass opacification (GGO) patterns (82%) as well as bilateral lung involvement (66%). Progression to acute respiratory distress syndrome (ARDS) was the most common complication of patients testing positive for COVID-19. We found 11/83 (13.2%) reports on Acute Respiratory Distress Syndrome (ARDS), 18 of 86 (21%) investigated cases had ARDS. Mortality outcomes were difficult to assess; only 10 studies showed mortality data in which 17/108 (16%) COVID-19 patients died. A wide range of therapeutic modalities was tried across studies, with antiviral treatments being the most used.

**Table 1:** Characteristics of the included studies.

**References**	**Country**	**Published time**	**Type of study**	**Mean age**	**Male/** **Female**	**No. of patient (s)**	**Diagnostic methods**
Kim et al. (8)	South Korea	19, Feb, 2020	Case report	45	1M,1F	2	RT-PCR/CT-scan
Yu et al. (9)	China	18, Feb, 2020	Case report	74.2	2M, 2F	4	RT-PCR
Bastola et al. (10)	Nepal	10, Feb, 2020	Case report	32	M	1	RT-PCR
Duan and Qin (11)	China	4, Feb, 2020	Case report	46	F	1	RT-PCR/CT-scan
Fang et al. (12)	China	19, Feb, 2020	Case report	47	M	1	RT-PCR/CT-scan
Han et al. (13)	China	19, Feb, 2020	Case report	47	M	1	RT-PCR/CT-scan
Wei et al. (14)	China	25, Feb, 2020	Case report	62	M	1	RT-PCR/CT-scan
Holshue et al. (15)	USA	5, Mar, 2020	Case report	35	M	1	RT-PCR
Lim et al. (16)	South Korea	14, Feb, 2020	Case report	54	M	1	RT-PCR/CT-scan
Shi et al. (17)	China	4, Feb, 2020	Case report	42	M	1	RT-PCR/CT-scan
Silverstein et al. (18)	Canada	13, Feb, 2020	Case report	56	M	1	RT-PCR
Wei et al. (19)	China	17, Feb, 2020	Case report	40	F	1	RT-PCR
Wu et al. (20)	China	3, Feb, 2020	Case report	41	M	1	RT-PCR
Xu et al. (21)	China	17, Feb, 2020	Case report	50	M	1	RT-PCR
Winichakoon et al. (22)	Thailand	26, Feb, 2020	Case report	28	M	1	RT-PCR
Zhan et al. (23)	China	28, Jan, 2020	Case report	38	1M, 1F	2	RT-PCR
Fang et al. (24)	China	7, Feb, 2020	Case report	38.5	1M,1F	2	RT-PCR/CT-scan
Lin et al. (25)	China	11, Feb, 2020	Case report	37	M	2	RT-PCR/CT-scan
Liu et al. (26)	Taiwan	12, Mar, 2020	Case report	51	1M, 1F	2	RT-PCR
Phan et al. (27)	Vietnam	27, Feb, 2020	Case report	Father: 65, Son: 27	M	2	RT-PCR
Pongpirul et al. (28)	Thailand	12, Mar, 2020	Case report	51	M	1	RT-PCR
Hao et al. (29)	China	2, Feb, 2020	Case report	60	M	1	RT-PCR/CT-scan
Hao and Li (30)	China	17, Feb, 2020	Case report	58	M	1	RT-PCR
Zhang et al. (31)	China	11, Feb, 2020	Case report	3 months	1M	1	RT-PCR
Bai et al. (32)	China	17, Feb, 2020	Case series	53.4	3M/4F	7	RT-PCR
Cai et al. (33)	China	4, Feb, 2020	Case report	7	1M	1	RT-PCR
Zeng et al. (34)	China	17, Feb, 2020	Case report	17 days	1M	1	RT-PCR
Chan et al. (35)	China	24, Jan, 2020	Case series	46	3M,3F	6	RT-PCR
Chen et al. (36)	China	12, Feb, 2020	Case series	29.8	F	9	RT-PCR/CT-scan
Wei et al. (37)	China	21, Feb, 2020	Case series	6 months	2 M, 7F	9	RT-PCR
Qin et al. (38)	China	22, Feb, 2020	Case series	55.5	2M, 2F	4	CT-scan
Wang et al. (39)	China	9, Feb, 2020	Case series	44.2	3M, 1F	4	RT-PCR/CT-scan
Xie et al. (40)	China	12, Feb, 2020	Case series	48.4	M4, F1	5	RT-PCR
Yoon et al. (41)	Korea	18, Feb, 2020	Case series	54	4M, 5F	9	CT-scan
Stoecklin et al. (42)	France	13, Feb, 2020	Case series	36.3	2M, 1F	3	RT-PCR
Rothe et al. (43)	Germany	5, Mar, 2020	Case series	33	NR	5	RT-PCR
Bai et al. (44)	China	21, Feb, 2020	Case series	42-57	1M, 5F	6	RT-PCR
Tong et al. (45)	China	9, May, 2020	Case series	31	4M, 3F	7	RT-PCR
Feng et al. (46)	China	16, Feb, 2020	Case series	7	5M/10F	15	RT-PCR
Zhang et al. (47)	China	15, Feb, 2020	Case series	36	5M/4F	9	RT-PCR
Liu et al. (48)	China	17, Feb, 2020	Case series	35	10M/20F	30	RT-PCR
Albarello et al. (49)	Italy	20, Feb, 2020	Case series	66.5	1M/1F	2	RT-PCR
Asadollahi-Amin et al. (50)	Iran	7, Apr, 2020	Case report	44	M	1	RT-PCR
Bhat et al. (51)	USA	11, Apr, 2020	Case series	54.5	6M/2F	8	RT-PCR
Chen et al. (52)	China	1, Apr, 2020	Case series	52.6	2M/1F	3	RT-PCR
Wang et al. (53)	China	9, Apr, 2020	Case series	42	11M/15F	26	RT-PCR
Liu et al. (54)	China	16, Apr, 2020	Case series	54	2M/1F	3	RT-PCR
Lu et al. (55)	China	19, Mar, 2020	Case series	NM	NM	3	RT-PCR
Lin et al. (56)	China	22, Feb, 2020	Case report	61	M	1	RT-PCR
Mousavi et al. (57)	Afghanistan	5, Apr, 2020	Case report	35	M	1	RT-PCR
Hamer et al. (58)	Germany	26, Mar, 2020	Case report	59	M	1	RT-PCR
Gupta et al. (59)	India	10, Apr, 2020	Case series	40.3	14M/7F	21	RT-PCR
Moreira et al. (60)	Brazil	3, Apr, 2020	Case report	73	M	1	RT-PCR
Gao et al. (61)	China	24, Mar, 2020	Case series	54.6	1M/2F	3	RT-PCR
Marchand-Senécal et al. (62)	Canada	9, Mar, 2020	Case report	56	M	1	RT-PCR
Lin et al. (25)	China	11, Feb, 2020	Case series	37	2M	2	RT-PCR
Makurumidze (63)	Zimbabwe	2, Apr, 2020	Case series	NM	2M/6F	8	RT-PCR
Li et al. (64)	China	7, Apr, 2020	Case series	8	12M/10F	22	RT-PCR
Li et al. (65)	China	6, Apr, 2020	Case report	74	F	1	CT-Scan
Li et al. (66)	China	30, Mar, 2020	Case series	61	13M/12F	25	RT-PCR
Cheng et al. (67)	Taiwan	16, Apr, 2020	Case report	55	F	1	RT-PCR
Edrada et al. (68)	Philippines	14, Apr, 2020	Case series	41.5	1M/1F	2	RT-PCR
Feng et al. (69)	China	7, Apr, 2020	Case report	34	M	1	CT-Scan
Woznitza et al. (70)	UK	2, Apr, 2020	Case series	78	1M/2F	3	RT-PCR
Zeng et al. (71)	China	5, Apr, 2020	Case report	63	M	1	RT-PCR
Zhang et al. (72)	China	18, Mar, 2020	Case report	64	M	1	RT-PCR
Zhou et al. (73)	China	3, Apr, 2020	Case series	NM	1M/3F	4	RT-PCR
Torkian et al. (74)	Iran	27, Mar, 2020	Case series	46	2M/1F	3	RT-PCR
Tan et al. (75)	China	3, Apr, 2020	Case series	7	3M/7F	10	RT-PCR
Hase et al. (76)	Japan	2, Apr, 2020	Case report	35	F	1	RT-PCR
Huang et al. (77)	Taiwan	19, Feb, 2020	Case series	73.7	2F	2	RT-PCR
Hu et al. (78)	China	4, Mar, 2020	Case series	32.5	8M/16F	24	RT-PCR
Hu et al. (78)	Italy	27, Mar, 2020	Case report	53	F	1	RT-PCR
Kim et al. (79)	South Korea	6, Apr, 2020	Case series	40	15M/13F	28	RT-PCR
Kim et al. (80)	South Korea	3, Feb, 2020	Case report	35	F	1	RT-PCR
Kong et al. (81)	South Korea	14, Feb, 2020	Case series	42.6	15M/13F	28	RT-PCR
Lee et al. (82)	Taiwan	10, Mar, 2020	Case report	46	F	1	RT-PCR
Lescure et al. (83)	France	27, Mar, 2020	Case series	47	3M/2F	5	RT-PCR
Wissenberg et al. (84)	Denmark	3, Apr, 2020	Case report	50	M	1	RT-PCR
Li et al. (85)	China	1, Mar, 2020	Case series	55	2M/1F	3	RT-PCR

**Table 2:** Summary of the case report and case series findings.

	**Variables**	**Number** **of studies**	n/N^*^	**%**
Comorbidities	Hypertension	22	44/228	19
	Cardiovascular disease	6	11/137	8
	Diabetes	17	27/241	11
	Pulmonary disease	8	13/107	12
Clinical manifestations	Fever	68	248/401	62
	Cough	39	195/389	50
	Dyspnea	30	78/279	28
	Myalgia/fatigue	38	106/343	31
	Sputum production	14	49/197	25
	Sore throat	20	48/164	29
	Headache	11	37/149	25
	Diarrhea	14	21/94	22
	Nausea/vomiting	8	17/84	20
	Dizziness	5	5/35	14
	Rhinorrhea	13	22/196	11
	Chills	4	4/13	31
Laboratory findings	Lymphopenia	24	83/185	45
	Leukopenia	17	38/150	25
	Thrombocytopenia	8	26/69	38
	High CRP	18	118/197	60
	High LDH	14	34/77	44
	High ESR	10	17/42	40
	High AST	11	23/48	48
	High ALT	13	22/77	28.5
	High creatinine kinase	8	9/44	20
	High creatinine	4	6/32	19
CT	Both of GGO and Consolidation	16	32/59	54
	GGO without consolidation	20	48/60	80
	Unilateral	11	35/87	40
	Bi lateral	23	76/110	69
Complications	ARDS	11	18/86	21
	Hospitalization	30	77/83	93
Outcomes	Discharged	23	137/205	67
	Death	10	17/108	16

* *n, number of patients with any variables; N, the total number of patients with COVID-19*.

Common antiviral treatment modalities included lopinavir (HIV protease inhibitor), arbidiol hydrochloride (influenza fusion inhibitor), and oseltamivir (neuraminidase inhibitor). In Table 3 we summarize all of the drugs used.

**Table 3:** Treatment agents used in the included studies.

	**Treatment**	**Agents**	**Number of studies**	n/N^*^	**%**
Pharmacologic treatment	Antiviral drugs	Lopinavir	6	9/9	100
		Arbidol hydrochloride	2	6/6	100
		Oseltamivir	5	1/1	100
		Veletonavir	1	1/1	100
		Remdesivir	1	1/1	100
		Ribavirin	1	1/1	100
		Ritonavir	1	1/1	100
		Gancyclovir	1	1/1	100
	Antibacterial drugs	Moxifloxacin	4	5/5	100
		Vancomycin	1	1/1	100
		Cefepime	1	1/1	100
		Meropenem	2	2/2	100
		Piperacillin tazobactam	2	2/2	100
		Sefoselis	1	1/1	100
		Linezolid	1	1/1	100
		Levofloxacin	1	1/2	50
	Others	Methylprednisolone	5	6/6	100
		Ambroxol Hydrochloride	1	1/1	100
		Acetaminophen	2	2/2	100
		Ibuprofen	2	2/2	100
		Intravenous Immunoglobulin	3	4/7	57
		Guaifenesin	1	1/1	100
		Ondansetron	1	1/1	100
		Interferon alpha-2b	2	2/2	100
		Herbal patent medicine	2	3/3	100
Non-pharmacologic treatment	Oxygen therapy	Non-invasive	6	10/10	100

* *n, number of patients under treatment; N, the total number of patients with COVID-19*.

## Discussion

The 2019 novel coronavirus has been declared a public health emergency worldwide. The World Health Organization (WHO) declared COVID-19 a pandemic affecting 110 countries around the world with a continued global spread. The 2019-nCoV is likely to be transmitted by asymptomatic individuals (86). Asymptomatic transfer leads to lower prevalence estimates and higher transmission rates in the community. Until universal screening and vaccination become available, it is necessary to trace close contacts of those testing positive for COVID-19 and quarantining contacts to prevent asymptomatic transmission.

According to the articles we included, 2019-nCoV can only be transferred from person to person (87). Chen et al. suggested that they had no evidence of vertical transmission from mother to child (36). Any person infected with 2019-nCoV can develop a clinical course of Covid-19. However, it is reported to cause the most severe symptoms such as respiratory failure in older men with comorbidities (88). Children, teenagers, and younger people mostly showed a mild presentation of the disease (89).

Based on our reviewed articles, hypertension, diabetes, cardiovascular disease, and pulmonary disease were the most common morbidities among COVID-19 patients. This point was also mentioned in Alraddadi et al. study about MERS-CoV patients (90). They showed that individuals with comorbidities like diabetes, smoking, and cardiovascular disease were associated with a more severe clinical course (90). According to Yang et al., chronic diseases can debilitate the immune system and make pro-inflammatory conditions, leading to more severe infection and subsequently higher mortality rates (91).

According to the included studies, the most common clinical manifestations were fever, cough, dyspnea, and myalgia or fatigue. Less common clinical manifestations included nausea or vomiting, dizziness, rhinorrhea, and chills. Liu et al. reported that infants had mild clinical manifestations and a better prognosis. Furthermore, some asymptomatic cases were seen among children.

The most common abnormal laboratory changes were lymphopenia, high concentrations of C-reactive protein, and elevated levels of aspartate aminotransferase; however, we do not know the exact pathogenesis and the reason for these alterations. Laboratory abnormalities may indicate the severity of disease and developing complications. According to Huang et al., most patients with secondary infection had a procalcitonin level >0.5 ng/Ml and ICU patients had higher levels of prothrombin time and D-dimer (92). Also, Liu et al. mentioned using hypoalbuminemia, lymphopenia, high concentrations of CRP, and elevated LDH to predict the severity of acute lung injury (3). Higher levels of angiotensin II are also proposed to be related to acute lung injury (3). Meanwhile, non-survivors are suggested to have higher D-dimer and FDP levels, longer PT and aPTT, and lower fibrinogen and antithrombin levels (93).

CT scan as a diagnostic tool can be used to evaluate the severity of pulmonary involvement and monitor clinical progression. CT scan has good sensitivity and can be used to establish COVID-19 diagnosis in patients who are highly suspicious based on epidemiologic history and clinical manifestations but have negative PCR-based test results (12, 94). It is important to highlight that the CT scan can be normal during initial days, and a normal CT scan in a suspected case would never definitely rule out the diagnosis of COVID-19 (95). Moreover, the CT scan is dynamic in patients with COVID-19 and changes rapidly (13, 17, 19). The earliest abnormal finding in the CT scan is the appearance of ground-glass opacities in peripheral and sub-pleural areas (96). As the disease progresses, the GGO's will expand and distribute more, most commonly to the right lower lung lobes. Later findings include consolidations, paving patterns, thickening of lobar fissures, and adjacent pleura. Pleural effusion, hilar lymphadenopathies, and mediastinal lymphadenopathies are not common findings and have only been reported scarcely (40). Lung pathology can progress to a “white lung” with low functional capacity or heal with some fibrotic remnants (40). Dynamic changes in the lungs seen on CT imaging will continue even after the patient's discharge (96). CT scan findings have prognostic value in some patients, as Shi et al. have reported, deterioration on follow-up CT scan, old age, male sex, and underlying comorbidities are associated with poor prognosis.

ARDS was the most common complication among the confirmed COVID-19 patients; the development of ARDS increased the risk of patient mortality (97). Huang et al. reported that the median time from onset of symptoms to the development of ARDS was 9 days (92). Other complications were acute cardiac injury, acute kidney injury, secondary infection, and shock that leads to multiple organ failure (98, 99). ICU patients in comparison to non-ICU patients were also more likely to have complications (100). The mortality rate was higher in critically ill patients as well as in older patients with comorbidities and ARDS. Yang et al. reported that the median duration from ICU admission to death was 7 days (97). The window between the presentation to the time of ICU admission and/or development of ARDS is an optimal time for medical intervention.

Also, the results of the current study are in comparison with the recent large patient cohort studies in the aspect of comorbidities, clinical manifestations, laboratory, and radiological findings, however, there are some differences (101, 102). In a study by Richardson et al., a more detailed analysis of the patient's vital signs, ICU interventions, outcome characteristics, and risk factors were reported (101). According to their study, among the patients who were discharged or had died during hospitalization, 14.2% were treated in the ICU, 12.2% received invasive mechanical ventilation, 3.2% were treated with kidney replacement therapy, and 21% died. Moreover, Grasselli et al. indicated that Older patients (age ≥ 64 years) had higher mortality than younger patients (age ≤ 63 years) (36%vs 15%) (102).

There are many challenges in COVID-19 therapeutic strategies. There is currently no cure for COVID-19. However, pharmacologic and non-pharmacologic symptom management and supportive care measures should be given to all patients with symptomatic COVID-19. Other various therapeutic strategies have been trialed in patients with COVID-19 to slow disease progression. There is a paucity of data surrounding the efficacy of treatments. Of the case controls and case series we included, antiviral agents including HIV protease inhibitors (lopinavir and ritonavir) as well as anti-influenza compounds (oseltamivir and arbidol) were used as treatment regimens. Unfortunately, we didn't have enough information about the efficacy of each regimen; however, according to some studies, anti-HIV based medications could have benefits in more rapid improvement of clinical manifestations and decrease in viral load (13, 16, 19).

A limitation of this review relates to the potential risk of bias. Bias occurs in the case reports/series studies because their results are not representative and do not represent the truth. A further limitation is that the conclusions are limited due to the case reports and case series. We did not include observational studies and randomized controlled trial (RCT)/quasi-randomized studies, because another study being conducted by the authors. Furthermore, the focus of the reviewed case reports and case series was mainly on the clinical description of the patients with COVID-19, but detailed information on the treatment outcomes and medical consequences were rarely provided. Also, the case number included in this systematic review is low compared with the currently published patient cohort, and this may lead to the declining clinical significance of this manuscript. Finally, our results are limited to younger adults who had been hospitalized during the 4–5 first months of the COVID-19 pandemic.

In conclusion, we discussed the clinical symptoms, laboratory abnormalities, common comorbidities, imaging modalities, and potential therapeutic options in COVID-19. We indicated that the most common symptoms were fever, cough, and dyspnea, but some young infected cases had no signs or symptoms. ARDS was the most common reported complication and was associated with poor prognosis. In the wake of the COVID-19 pandemic, countries are scrambling to produce enough RT-PCR diagnostic tests. Diagnostic information from other surrogate markers would be valuable if markers proved to be sensitive and specific. Namely, we learned that laboratory data like CRP may not only be related to the severity of the disease, but it may be predictive of disease outcomes. Further studies are needed to relate quantified elevations in CRP to disease severity. Due to the high sensitivity of the CT scan, it is considered as a good diagnostic tool. However, it should be kept in mind that a normal CT scan will never rule out the diagnosis of COVID-19 in a highly suspicious case based on history and clinical findings. Lastly, there are different therapeutic strategies for COVID-19 patients, but we don't have enough data for their efficacy. Additional investigations including randomized controlled trials will be necessary to further our understanding of the treatment of COVID-19.

## Data Availability Statement

All datasets presented in this study are included in the article/ supplementary material.

## Author Contributions

MN and MM designed the study and revised the manuscript. MN, AT, MA, YF, PJ, SH, and TC performed the search, data extraction, statistical analysis, and wrote the first draft of the manuscript.

## Conflict of Interest

The authors declare that the research was conducted in the absence of any commercial or financial relationships that could be construed as a potential conflict of interest.
